# A Major Role for Mammals in the Ecology of *Mycobacterium ulcerans*

**DOI:** 10.1371/journal.pntd.0000791

**Published:** 2010-08-10

**Authors:** Janet A. M. Fyfe, Caroline J. Lavender, Kathrine A. Handasyde, Alistair R. Legione, Carolyn R. O'Brien, Timothy P. Stinear, Sacha J. Pidot, Torsten Seemann, M. Eric Benbow, John R. Wallace, Christina McCowan, Paul D. R. Johnson

**Affiliations:** 1 Victorian Infectious Diseases Reference Laboratory, North Melbourne, Victoria, Australia; 2 WHO Collaborating Centre for Mycobacterium ulcerans, Victorian Infectious Diseases Reference Laboratory, North Melbourne, Victoria, Australia; 3 Department of Zoology, The University of Melbourne, Parkville, Victoria, Australia; 4 Faculty of Veterinary Science, The University of Melbourne, Parkville, Victoria, Australia; 5 Department of Microbiology and Immunology, The University of Melbourne, Parkville, Victoria, Australia; 6 Victorian Bioinformatics Consortium, Monash University, Clayton, Victoria, Australia; 7 Department of Biology, University of Dayton, Dayton, Ohio, United States of America; 8 Department of Biology, Millersville University, Millersville, Pennsylvania, United States of America; 9 Faculty of Veterinary Science, The University of Melbourne, Werribee, Victoria, Australia; 10 Infectious Diseases Department, Austin Health, Heidelberg, Victoria, Australia; Swiss Tropical Institute, Switzerland

## Abstract

**Background:**

*Mycobacterium ulcerans* is the causative agent of Buruli ulcer (BU), a destructive skin disease found predominantly in sub-Saharan Africa and south-eastern Australia. The precise mode(s) of transmission and environmental reservoir(s) remain unknown, but several studies have explored the role of aquatic invertebrate species. The purpose of this study was to investigate the environmental distribution of *M. ulcerans* in south-eastern Australia.

**Methodology/Principal Findings:**

A range of environmental samples was collected from Point Lonsdale (a small coastal town southwest of Melbourne, Australia, endemic for BU) and from areas with fewer or no reported incident cases of BU. *Mycobacterium ulcerans* DNA was detected at low levels by real-time PCR in soil, sediment, water residue, aquatic plant biofilm and terrestrial vegetation collected in Point Lonsdale. Higher levels of *M. ulcerans* DNA were detected in the faeces of common ringtail (*Pseudocheirus peregrinus*) and common brushtail (*Trichosurus vulpecula*) possums. Systematic testing of possum faeces revealed that *M. ulcerans* DNA could be detected in 41% of faecal samples collected in Point Lonsdale compared with less than 1% of faecal samples collected from non-endemic areas (p<0.0001). Capture and clinical examination of live possums in Point Lonsdale validated the accuracy of the predictive value of the faecal surveys by revealing that 38% of ringtail possums and 24% of brushtail possums had laboratory-confirmed *M. ulcerans* skin lesions and/or *M. ulcerans* PCR positive faeces. Whole genome sequencing revealed an extremely close genetic relationship between human and possum *M. ulcerans* isolates.

**Conclusions/Significance:**

The prevailing wisdom is that *M. ulcerans* is an aquatic pathogen and that BU is acquired by contact with certain aquatic environments (swamps, slow-flowing water). Now, after 70 years of research, we propose a transmission model for BU in which terrestrial mammals are implicated as reservoirs for *M. ulcerans*.

## Author Summary

*Mycobacterium ulcerans* is the causative agent of Buruli ulcer (BU), a destructive skin disease found predominantly in sub-Saharan Africa and south-eastern Australia. The mode of transmission and environmental reservoir remain unknown, but several studies have explored the role of aquatic insects, such as water bugs, and biting insects, such as mosquitoes. In the present study we investigated possible environmental source(s) of *M. ulcerans* in Victoria, Australia. Our results revealed that although *M. ulcerans* DNA could be detected at low levels in a variety of environmental samples, the highest concentrations of *M. ulcerans* DNA were found in the faeces of two species of possums, common ringtails and common brushtails. Possums are small arboreal marsupial mammals, native to Australia, and these particular species occur in both urban and rural areas. Examination and sampling of live captured possums in an area endemic for BU revealed that 38% of ringtail possums and 24% of brushtail possums, respectively, had laboratory-confirmed *M. ulcerans* lesions and/or *M. ulcerans* PCR-positive faeces. The finding that large numbers of possums in a BU-endemic area are infected with *M. ulcerans* raises the possibility that mammals are an environmental reservoir for *M. ulcerans*.

## Introduction

Buruli ulcer (BU) is caused by the environmental mycobacterium, *Mycobacterium ulcerans*. Infection with *M. ulcerans* often leads to extensive necrosis of the skin and soft tissue with the formation of large ulcers, usually on the leg or arm, due to the production of the destructive polyketide toxin, mycolactone [1]. Although rarely fatal, BU causes serious morbidity and frequently results in permanent disability [2]. The disease has been reported in more than 30 countries worldwide; however, cases mainly occur in regions with tropical and subtropical climates. The majority of cases are found in West and sub-Saharan Africa. Cases of BU often cluster around particular water bodies and are highly focally distributed, with endemic and non-endemic communities often separated by only a few kilometres [2].

**Figure 1:**
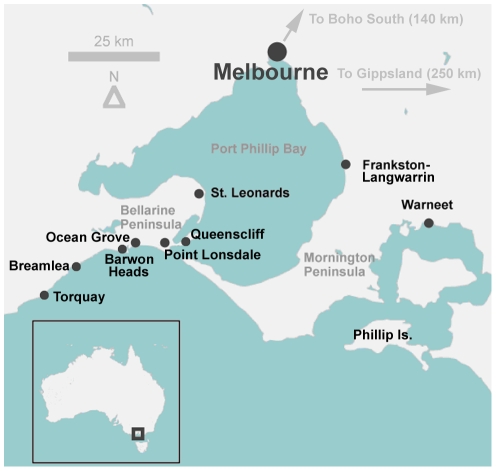
Map of central coastal Victoria, showing places referred to in the text or associated references.

Australia is the only developed country reporting significant local transmission of *M. ulcerans*. In 1948, a cluster of cases linked to the Bairnsdale region in Gippsland was described by McCallum *et al.* [3]. Since then, foci of infection have been reported in tropical far north Queensland [4] and temperate coastal Victoria, where there have been several outbreaks over the past two decades: Phillip Island (1992–1995), the Frankston/Langwarrin region (1990–1997), St Leonards (2001–2002) and Point Lonsdale (2002-present) (Fig. 1) [5], [6]. The present outbreak in Point Lonsdale, a small coastal town approximately 60 km south-west of the Victorian capital Melbourne, is the largest on record in Australia, with over 100 laboratory-confirmed cases diagnosed since 2002. Geographically, the town is close to sea level, and there are several natural and man-made swamps and water features in the area [6]. Cases of BU have also been described in both native wildlife and domestic mammal species in Victoria, including koalas (*Phascolarctos cinereus*) [7], common ringtail possums (*Pseudocheirus peregrinus*) [8], a mountain brushtail possum (*Trichosurus cunninghami*), a long-footed potoroo (*Potorous longipes*) (J. Fyfe, unpublished), two horses [9], two dogs (O'Brien *et al.*, manuscript in preparation), an alpaca [8] and a cat [10]. All animal cases were identified in locations where human cases of BU have been reported.

The precise mode(s) of transmission and environmental reservoir(s) of BU are unresolved and continue to be the subject of intense research. Proximity to marshes and wetlands is a recognised risk factor for infection and several studies have explored the role of aquatic invertebrate species as potential vectors and/or reservoirs [6], [11]–[13]. Detection of *M. ulcerans* in environmental samples is mainly achieved using PCR, as culturing *M. ulcerans* directly from the environment is extremely difficult [14]. In Australia, *M. ulcerans* DNA was detected in water and detritus from swamps during the outbreak of BU on Phillip Island in the mid-1990s [15], [16] and more recently in five species of mosquitoes (*Aedes* sp., *Coquillettidia* sp. and *Culex* sp.) captured from Point Lonsdale (infection rate, 4.3/1,000 mosquitoes) [6]. In West Africa, *M. ulcerans* DNA has been detected in water and aquatic plants [17], insects (Belastomatidae, Naucoridae, Hydrophilidae), crustaceans and molluscs (*Bulinus* sp. and *Planorbis* sp.) and small fish (including *Tilapia* sp.) [11], [13], [18]–[21]. Recent studies of the distribution of *M. ulcerans* in aquatic sites in Ghana found evidence of *M. ulcerans* DNA in insects, water filtrate, biofilm and soil [12], [13]. In 2008, Portaels *et al.* described, for the first time, the cultivation and characterisation of an *M. ulcerans* strain obtained from an aquatic Hemiptera (common name Water Strider, *Gerris* sp.) from Benin [14].

Analysis of the whole genome sequence of *M. ulcerans* has provided further insights into the elusive environmental reservoir and mode of transmission [22]. Complete sequencing of an *M. ulcerans* strain isolated from a patient in Ghana revealed a 5,631,606 bp circular chromosome with 4160 genes, 771 pseudogenes and a 174,155 bp virulence plasmid pMUM001 that is required for the production of mycolactone [23], [24]. Comparison of the *M. ulcerans* genome with the genome of *M. marinum* confirmed the very close relationship between these species; however, it also revealed that there are some striking differences, mostly due to the presence of the plasmid pMUM001 and the many chromosomal deletions and rearrangements that have occurred in *M. ulcerans* [23]. It is therefore likely that *M. ulcerans* has evolved from an *M. marinum*-like ancestor by lateral gene transfer and reductive evolution, through the acquisition of a pMUM001-like plasmid, expansion of the two high copy number insertion sequence elements IS*2404* and IS*2606*, extensive gene disintegration (formation of pseudogenes), genome rearrangements and DNA deletion. These characteristics suggest that *M. ulcerans* has recently passed through a so-called “evolutionary bottleneck” and is adapting to a new, niche environment.

In this study, we investigated potential environmental reservoirs of *M. ulcerans* in south-eastern Australia with the aim of developing a more comprehensive model of its life cycle and mode of transmission. Specifically, using semi-quantitative real-time PCR and culture to test for the presence of *M. ulcerans*, we investigated a range of potential abiotic and biotic reservoirs (selected using emerging information in the literature and our own ongoing field based research) in areas of varying BU endemicity. Our findings have led us to propose that *M. ulcerans* is able to infect small mammals, survive and potentially replicate within their gastrointestinal tracts and raises the possibility that mammals play a major role in the ecology of *M. ulcerans*.

## Materials and Methods

### Environmental samples

#### a. Study sites and sample collection

This study was conducted in Victoria, Australia, primarily at Point Lonsdale on the Bellarine Peninsula (a current human BU outbreak zone, and therefore classified as endemic). A number of other sites, classified as areas of low endemicity (where BU infection has occurred in the past or fewer cases have been recorded recently), or non-endemic (no recorded human or animal BU cases), were also sampled (Fig. 1). The number and types of samples collected and tested are shown in Tables 1 and 2. Following collection, all samples were stored in sterile plastic containers or zip-lock bags, transported cool to the laboratory and stored at 4°C prior to DNA extraction, usually within a week of collection.

**Table 1:** Detection of *M. ulcerans* DNA (IS*2404*, IS*2606* and KR) in environmental samples collected from Point Lonsdale (endemic) and sites of low endemicity in Victoria, Australia.

*Sample type*	*No. samples positive/no. samples tested*			
	*Point Lonsdale* a	*Bellarine Peninsula* b	*Phillip Island* c	*Gippsland* d
Suspended solids/water residue	4/4 (100%)^e^	0/10	0/9	0/10
Aquatic plant biofilm	2/10 (20%)	0/5	0/2	0/2
Aquatic plants	1/9 (11%)	0/5	0/5	0/2
Aquatic macroinvertebrates	0/12	0/15	0/4	0/7
Detritus	3/14 (22%)	-	-	0/33
Sediment	9/27 (33%)	0/1	-	-
Soil	22/36 (61%)	2/7 (29%)	0/3	0/3
Terrestrial Plants	9/51 (18%)	0/3	0/4	2/21 (10%)
Brushtail possum faeces^f^	2/5 (40%)	-	0/5	-
**Total**	**52/168 (32%)**	**2/51 (4%)**	**0/32**	**2/78 (3%)**

a High endemicity area.

b Ocean Grove, Queenscliff, St Leonards (low endemicity areas).

c Low endemicity area.

d Bellbird Creek, Sale (low endemicity areas).

e All four samples collected from the same site in Point Lonsdale on the same day.

f Preliminary testing only (see Table 2 for results of large scale testing).

**Table 2:** Detection of *M. ulcerans* DNA in possum faeces collected from BU high-, low- and non-endemic locations, in Victoria, Australia.

*Location*	*Total human BU cases, past 5 years* c	*Average annual incidence per 1000 population, past 5 years* d *(range)*	*Detection of M. ulcerans DNA in faeces by PCR* f			
			*Ringtail possum*		*Brushtail possum*	
			*No. positive/No. tested (%)*	*Median est. bacterial load* e	*No. positive/No. tested (%)*	*Median est. bacterial load* e
**High endemicity**						
Point Lonsdale	81	4.04 (0.81–8.07)	70/164 (43%)	10^4^	8/28 (29%)	10^2^–10^3^
**Low endemicity**						
Barwon Heads^a^	15	0.87 (0.00–2.00)	44/171 (26%)	10^4^	15/78 (19%)	10^2^–10^3^
Ocean Grove	11	0.18 (0.00–0.44)	0/29 (0%)		0/9 (0%)	
Queenscliff	6	0.85 (0.00–2.12)	3/43 (7%)	10^2^–10^3^	0/0	
Phillip Island	3	0.00	10/90 (11%)	10^2^–10^3^	1/76 (1%)	10^2^–10^3^
**Non-endemic**						
Boho South	0	0.00	0/29 (0%)		0/1 (0%)	
Breamlea	0	0.00	0/16 (0%)		0/0	
Greater Melbourne^b^	0	0.00	0/15 (0%)		0/43 (0%)	
Torquay	0	0.00	1/24 (4%)	10^2^–10^3^	0/7 (0%)	

a Appears to be an area of increasing BU endemicity, with seven of the 15 cases diagnosed in 2009.

b Comprises metropolitan suburbs of Clifton Hill, Clayton and Parkville.

c Laboratory-confirmed human cases in residents and visitors, 2005–09.

d Laboratory-confirmed human cases in residents only, 2005–09.

e Expressed as organisms/gram of faeces.

f All samples positive for IS*2404*. Subsets from each location were confirmed by IS*2606* and KR PCR.

#### b. Sampling methods

Aquatic environments were sampled for suspended solids/water residue collected from natural and man-made water bodies in Point Lonsdale and low endemicity sites. Two hundred millilitres (ml) of water was passed through a 1.6 micron fibreglass filter (Whatman Inc.) using a hand pump and/or 60–120 ml water through a 1.6 micron fibreglass filter (Whatman Inc.) using a syringe (volume was dependent on turbidity). Aquatic plant biofilms were collected from the dominant macrophytes (plant species) in natural and man-made water bodies, in Point Lonsdale and low endemicity areas, by placing the macrophyte samples in sterile bags, mixing with 200 ml clean water and scrubbing by hand to remove the biofilm. A 50 ml subsample was retained for each. A section of the stem from each macrophyte was also sampled. Aquatic macroinvertebrates were collected by sweeping a handheld D-frame aquatic net through a section of the water body for 45 seconds. Detritus, sediment and soil samples were collected from terrestrial and riparian sites using a hand held plastic sieve or by placing samples directly into a sterile container. Samples from terrestrial vegetation (leaves, bark, flowers, seeds etc) were collected and identified by botanist Neville Walsh (Senior Conservation Botanist, Royal Botanic Gardens, Melbourne).

**Figure 2:**
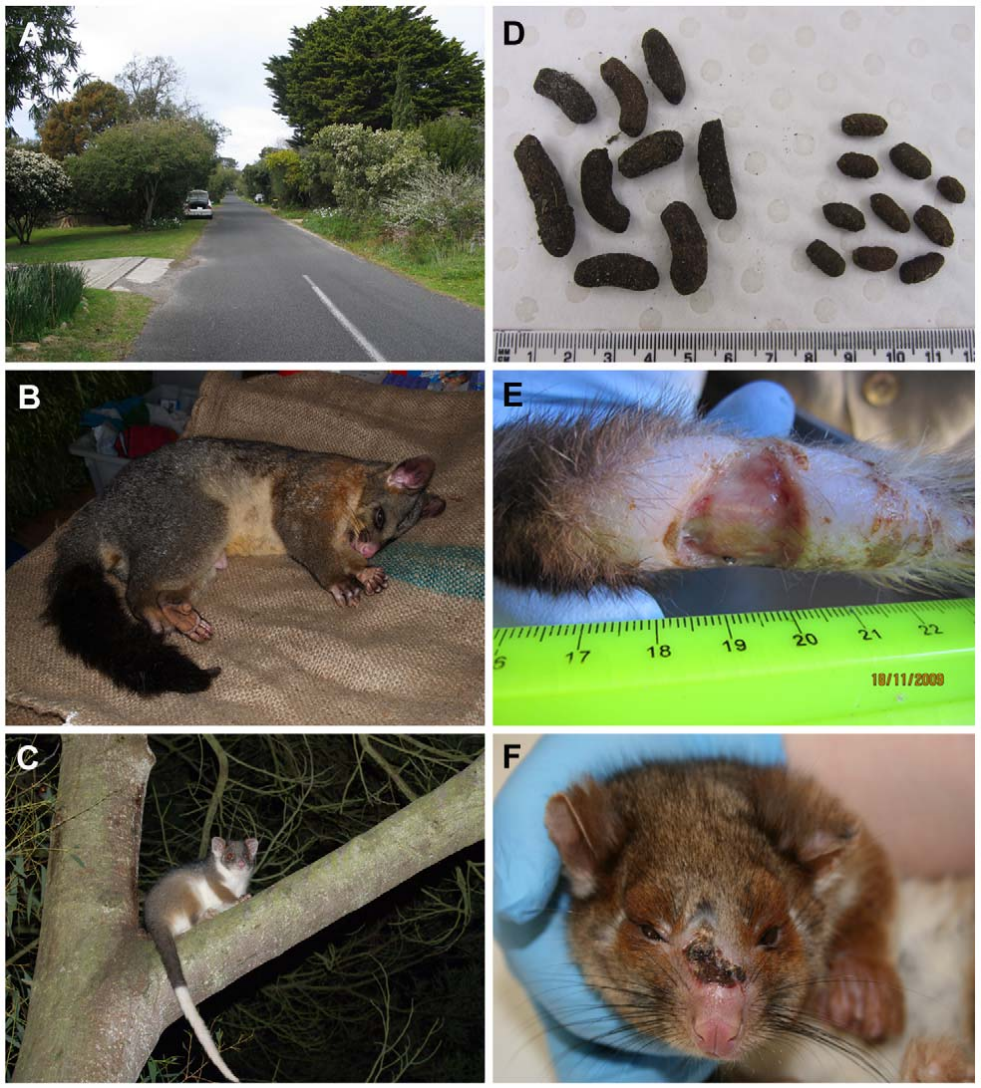
Photographs of Point Lonsdale, common brushtail possums and common ringtail possums. A. Point Lonsdale streetscape showing typical possum habitat. B. Common brushtail possum. C. Common ringtail possum. D. Brushtail possum faeces (left) and ringtail possum faeces (right). E. Ringtail possum tail lesion. F. Ringtail possum nose lesion.

Faecal samples from common ringtail possums and common brushtail possums (henceforth referred to as ringtail and brushtail possums) were collected directly from the ground, from the branches of trees or from fences, at 100- or 500-metre intervals along transects across areas of varying BU endemicity: Point Lonsdale (high endemicity area); Barwon Heads, Phillip Island, Ocean Grove and Queenscliff (low endemicity areas); Breamlea, metropolitan Melbourne, Boho South and Torquay (non-endemic areas) (Fig. 1). These sampling intervals were chosen to avoid any chance of repeated sampling from the same individual and were based on an estimated home range diameter for ringtail possums of no more than 100 metres (A. Legione, unpublished data). The identity of the animal host was determined by visual identification of the faecal sample (Fig. 2D), by an experienced zoologist (one of the authors) or with the aid of a scat and tracking manual [25].

### Live animal studies

#### a. Capture and sampling of live possums

The capture of possums, which are nocturnal, was based on standard operating procedures for the handling of wildlife developed by Dr Kath Handasyde, approved by The University of Melbourne Faculty of Veterinary Science Animal Experimentation Ethics Committee (project no. 0706769) and under permit from the Victorian Department of Sustainability and Environment (DSE permit no. 10004406). Cage traps, designed for live capture of brushtail and ringtail possums, baited with an apple smeared with peanut butter or a bait ball of peanut butter and rolled oats, were set 1–2 hours before dark in public and private properties throughout Point Lonsdale and then checked, commencing at dawn, the following morning. Ringtail possums were also caught at night, directly by hand, using a specifically designed noosing pole and a hand-held net. After capture, animals were transferred into material bags, and transported by car to a quiet, enclosed area, awaiting collection of samples and data.

#### b. Collection of samples and data from live possums

To minimise distress during handling and sampling, possums were heavily sedated with an I.M. injection of Zoletil (Virbac Australia Pty Ltd, 5–8 mg/kg) using a 29 gauge needle. Possums were examined for external lesions resembling BU and, if present, a specimen was obtained by swabbing the affected area. Faecal specimens, along with a number of other clinical samples that are described in a separate report (manuscript in preparation), were also collected. Animals were individually marked, via a number tattooed onto the ear, and a small PIT (passive induction transponder) tag, inserted subcutaneously between the shoulder blades, so that they could be identified in the event of recapture. Individual animals were subjected to full handling only once during any particular field trip. After handling and sampling, animals were placed individually into material bags, and held in an animal box in a quiet enclosed area. Animals were then released at dusk, on the same day, at the site of capture. However, in the circumstance that a captured animal was deemed, by a veterinarian, to be too unwell to be released, there was a provision for the animal to be euthanased using an overdose of pentobarbitone (150mg/kg).

### DNA-based analyses

#### a. DNA preparation

DNA was extracted from samples using the FastDNA® SPIN Kit for Soil with the FastPrep® Instrument (Qbiogene, Inc., Carlsbad, CA), after the following sample-dependent pre-extraction procedures: For soil, sediment, vegetation and possum faeces, ∼50–100 mg of wet or dry sample was directly added to the FastPrep Lysing Matrix E tube. Biofilm samples were prepared by centrifuging the Falcon tubes containing the 50 ml subsample at maximum speed for 10 mins. After removing the supernatant, the pellet was resuspended in kit-supplied Sodium Phosphate Buffer and transferred to the Lysing Matrix E tubes. Water residue was prepared by cutting the fibreglass filters into small pieces using a sterile scalpel and adding directly to the Lysing Matrix E tubes. Swabs were placed in sterile bead bottles with 2 ml phosphate buffered saline (PBS), vortexed, and 1 ml added to the Lysing Matrix E tubes. The Lysing Matrix E tubes were then centrifuged at maximum speed for 10 mins and the supernatant removed. After the sample-dependent pre-extraction procedures, DNA extraction was then performed according to the manufacturer's recommendations. DNA preparations were stored at −20°C.

#### b. Detection of *M. ulcerans* DNA

DNA extracts were tested for the presence of *M. ulcerans* DNA using two semi-quantitative real-time PCR assays targeting the insertion sequences IS*2404* and IS*2606* and a sequence encoding the ketoreductase B domain, KR, within the *mls*A1, *mls*A2 and *mls*B genes. These assays were developed and validated for use on environmental samples by Fyfe *et al.* [26] and are able to distinguish between *M. ulcerans* and other mycolactone-producing mycobacteria (MPM) that contain IS*2404*, but fewer copy numbers of IS*2606*, based on the difference in cycle threshold values between IS*2606* and IS*2404* (Δ*C_T_* [IS*2606-*IS*2404*]) [26]. All extracts were initially screened singly for the high copy number insertion sequence IS*2404*. This assay was multiplexed with an internal positive control to monitor PCR inhibition. Inhibited extracts were diluted 1/5 or 1/10 and repeat PCR performed. Extracts that were still inhibited at 1/10 dilution were omitted from analyses. With the exception of the possum faecal samples, all of the IS*2404*-positive DNA extracts from each sample type were tested in duplicate for IS*2606* and KR. In view of the large number of IS*2404*-positive DNA extracts from possum faecal samples obtained, a subset of these, taken from each of the different locations, was similarly confirmed. The Δ*C_T_* (IS*2606*-IS*2404*) were calculated to confirm that the sequences detected were attributable to *M. ulcerans* and not another MPM. To exclude the possibility of contamination, at least one negative control was included in every DNA extraction run, and four negative controls included in every real-time PCR assay.

#### c. Estimation of *M. ulcerans* bacterial loads in different samples

To estimate the *M. ulcerans* bacterial loads (expressed as *M. ulcerans*/gram or *M. ulcerans*/ml) in various sample types, the *C_T_* values obtained for IS*2404* were compared with a standard curve generated using a series of DNA extracts prepared from environmental samples that had been spiked with known numbers of *M. ulcerans* organisms [26]. These estimates were determined to provide an indication of the relative numbers of *M. ulcerans* between samples, rather than a strict quantitation of the number of organisms present in a sample, and hence are generally expressed as a 10-fold range.

#### d. Variable Number Tandem Repeat (VNTR)/Mycobacterial Interspersed Repeat Unit (MIRU) typing

VNTR/MIRU typing was performed using the conditions described previously [27]–[29] in 25 µl reactions using 1 µl of DNA template. PCR products were visualised on a 2% agarose gel and PCR product sizes estimated by comparing fragment sizes with a 100 bp DNA ladder (Promega, Wisconsin, USA). Products of the expected size were purified using a Roche High Pure PCR Purification Kit (Roche Diagnostics, Australia) and sequenced.

#### e. DNA sequence analysis

Sequence analysis of purified PCR products was performed using the BigDye ^(R)^ Terminator v3.1 Cycle Sequencing Kit (Applied Biosystems, Foster City, CA) according to the manufacturer's instructions. Reactions were analysed on an Applied Biosystems 3730S Genetic Analyzer (Applied Biosystems). Sequence data were edited using Bionumerics v4.0 (Applied Maths BVBA, Ghent, Belgium) and then compared with those derived from an *M. ulcerans* isolate, cultured from a human patient from Point Lonsdale.

#### f. Whole genome sequencing and assembly

Genomic DNA was prepared from a possum *M. ulcerans* isolate (JKD8170) and a human *M. ulcerans* isolate (JKD8049), both from Point Lonsdale. Whole genome sequencing was performed using an Illumina Genome Analyzer II with 36 cycle paired-end chemistry. Reads were mapped to the reference strain *M. ulcerans* Agy99 (GenBank accession CP000325) using SHRiMP [30]. Single nucleotide polymorphisms (SNPs) and micro-indels (DIPs) were detected using Nesoni, a software tool for analysing high-throughput DNA sequence data (used in [31]). Nesoni tallied the raw base counts at each mapped position in each of the reference strains, and then compared them using Fisher's Exact Test to find variable nucleotide positions in JKD8170 relative to JKD8049. To exclude the possibility that additional mutations in JKD8049 may have occurred in regions not present in the reference *M. ulcerans* Agy99, *de novo* assembly of JKD8170 and JKD8049 was performed using Velvet [32] and the above SNP/DIP detection procedure was repeated using the resulting contigs as reciprocal reference sequences. The read data for JKD8170 and JKD8049 have been deposited in the NCBI Sequence Read Archive (SRA) as part of Study accession number SRP001289.

### Culture of *M. ulcerans*

#### a. Culture of *M. ulcerans* from environmental samples

Culture of *M. ulcerans* from possum faeces was attempted by homogenising samples in bead bottles with Ringer's solution, decontaminating with an equal volume of 4% sodium hydroxide, incubating at room temperature for 15 mins and neutralising with 10% orthophosphoric acid (modified Petroff method). Samples were centrifuged at 4000 rpm for 20 mins and pellets resuspended in 2 ml Ringer's solution. 400 µl of the decontaminated suspension was used to inoculate Mycobacteria Growth Indicator Tube (MGIT) broths with PANTA added according to the manufacturer's recommendations (BD, Franklin Lakes, N.J.), Brown and Buckle slopes and 7H10 slopes with antibiotics (25 µg/ml piperacillin, 50 µg/ml amphotericin, 25 µg/ml vancomycin, 800 µg/ml actidione, 4 µg/ml aztreonam). MGIT broths and solid media were incubated at 31°C and monitored weekly for up to 16 weeks.

#### b. Culture of *M. ulcerans* from possum lesions

Swabs were placed in bead bottles with 2 ml phosphate buffered saline (PBS), vortexed, decontaminated with 2% sodium hydroxide, incubated at room temperature for 15 mins and neutralised with 10% orthophosphoric acid. Samples were then centrifuged at 4000 rpm for 20 mins and pellets resuspended in 2 ml Ringer's solution. 400 µl was used to inoculate Brown and Buckle slopes and MGIT broths with PANTA added according to the manufacturer's recommendations (BD, Franklin Lakes, N.J.) and were incubated at 31°C with weekly monitoring for up to 12 weeks.

### Human case definition and BU incidence

A case of BU was defined as a human patient with a suggestive clinical lesion from which *M. ulcerans* was identified by PCR [26] or culture from January 2005 to December 2009 inclusive. The likely geographic origin of infection was determined on the basis of the patient's residential address and/or travel history. A patient was considered as having acquired BU from a particular geographic area if he/she was a resident of, or a visitor to, that area and had not reported recent contact with any other known BU endemic area. Due to the large seasonal fluctuations in the population of endemic areas (most of which are summer holiday destinations), and the difficulty in estimating the number of visitors to a particular area, the average annual incidence of BU in each geographic area over the five-year study period was calculated by dividing the average annual number of cases in residents only (that is, cases in visitors were excluded) by the resident population of the specified geographic area. Resident population numbers were obtained using Australian Bureau of Statistics data derived from the 2006 Census of Population and Housing [33].

### Statistical analyses

Statistical analyses were performed using STATA version 10.0 (STATA Corporation, College Station, TX). Proportions were compared using the two-sample test of proportion.

## Results

### Environmental testing in Point Lonsdale and areas of low BU endemicity

Testing of environmental samples commenced in mid-2004, just prior to the peak of the Point Lonsdale outbreak. The initial focus was low-lying, wet areas in which mosquitoes were likely to breed, such as drains, soak pits (covered concrete pits into which storm water and street runoff flows and sits until it gradually seeps into the ground), man-made lakes and natural water bodies. In Point Lonsdale, low levels of *M. ulcerans* DNA (that is, weak positive real-time PCR signals for IS*2404*, IS*2606* and KR) were detected in sediment from a man-made lake; soil, sediment and detritus from a number of different soak pits and drains; biofilm; aquatic plants; and residue from filtered water (Table 1). The estimated bacterial loads for these samples ranged from 10–100 organisms/ml for residue from filtered water and 10^3^–10^4^ organisms/gram for biofilm. In contrast, only four samples (two soil and two vegetation) from low endemicity areas were positive for *M. ulcerans* DNA (Table 1).

In late 2006, the scope of our environmental testing expanded to samples in dryer areas at higher elevations, including leaf litter, leaves, tree bark, flowers, seeds, stems and faeces from brushtail possums (Table 1). The rationale for this was: (i) soil collected *outside* drains had previously tested positive for *M. ulcerans* DNA, (ii) BU patients have reported an association between small penetrating injuries, sustained from vegetation, and subsequent ulcers [34], and (iii) cases of BU are known to occur in arboreal marsupial mammals, including koalas [7] and ringtail possums [8]. Testing revealed that while *M. ulcerans* DNA could be detected at low levels in some samples of leaf litter and bark from trees (estimated bacterial load 10^2^–10^3^ organisms/gram), much higher levels of *M. ulcerans* DNA were detected in brushtail possum faeces (estimated bacterial load ≥10^6^ organisms/gram). This important discovery led to the large scale, systematic testing of possum faeces in Point Lonsdale, as well as low and non-endemic sites.

### Possum faecal testing in BU high-, low- and non-endemic sites

Over a two-year period (2007–09), systematic collection of faeces from brushtail and ringtail possums was carried out across Point Lonsdale, nearby low endemicity areas and non-endemic areas (Table 2). A total of 589 faecal samples from ringtail possums and 250 samples from brushtail possums were tested. The difference in the number of samples collected from each geographic location and from each species reflected the relative population densities, with ringtail possums being much more abundant than brushtail possums in many areas sampled (K. Handasyde and A. Legione, unpublished data).

**Figure 3:**
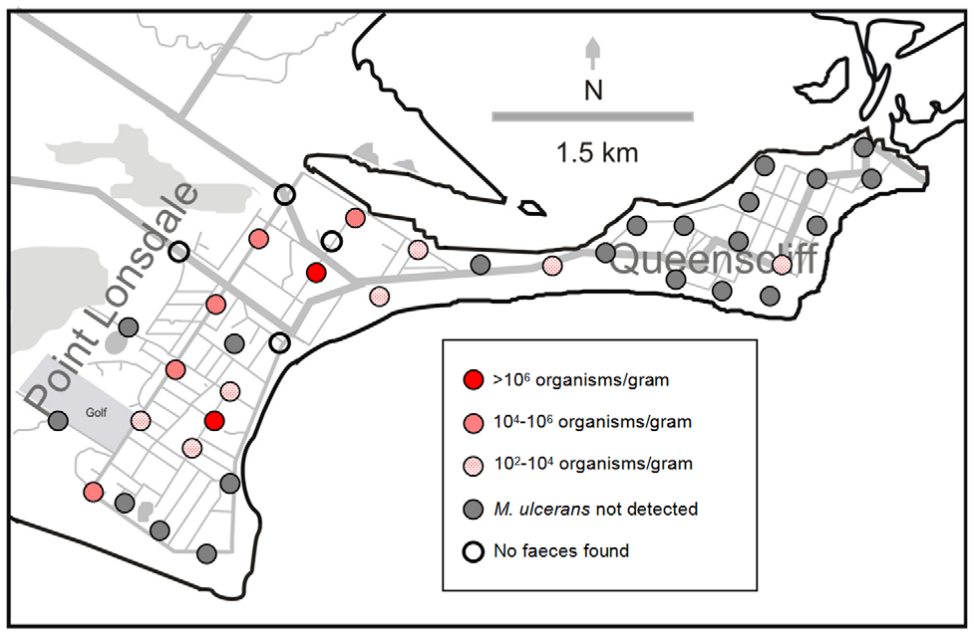
Distribution and estimated bacterial load of *M. ulcerans*-positive ringtail faecal samples in two towns. Map shows results of faecal surveys conducted in Point Lonsdale (approx. 81 human cases 2005–09) in August 2008 and Queenscliff (approx. 6 human cases 2005–09) in November 2008.

In Point Lonsdale, *M. ulcerans* DNA (IS*2404*) was detected in 43% of ringtail possum and 29% of brushtail possum faecal samples (Table 2). All samples tested for the presence of IS*2606* and KR were PCR-positive for these additional targets. Furthermore, the Δ*C_t_* (IS*2404*-IS*2606*) was always in the range expected for *M. ulcerans* (2.17–2.79), rather than another MPM (6.94–8.07) [26]. The median estimated bacterial load was 10^4^ organisms/gram (range: 10^2^–10^8^ organisms/gram) for ringtail possums (Fig. 3), with 17% of positive samples having an estimated bacterial load >10^6^ organisms/gram. The median estimated bacterial load for brushtail possum faeces was 10^2^–10^3^ organisms/gram (range: 10^2^–10^6^ organisms/gram).

In low endemicity areas, the proportion of PCR-positive faecal samples varied by location. For example, in Barwon Heads, where 15 human cases of BU have been reported since 2005, the proportion of positive ringtail and brushtail faecal samples was relatively high (26% and 19% respectively) compared with the other locations where fewer cases of BU have been reported (Table 2). The median estimated bacterial load of positive faecal samples from low endemicity areas also varied. In Barwon Heads the median estimated bacterial load for ringtail possum faeces was 10^4^ organisms/gram (with 16% of the positive samples having an estimated bacterial load >10^6^ organisms/gram). As in Point Lonsdale, the estimated bacterial load of the positive brushtail possum faeces in Barwon Heads was generally lower than for the ringtail possum faeces, with a median estimate of 10^2^–10^3^ organisms/gram. Similarly low *M. ulcerans* bacterial loads of 10^2^–10^3^ organisms/gram were estimated for faeces (ringtail possum only) collected in Queenscliff [Fig. 3] and Phillip Island. Only one sample collected from a non-endemic area (Torquay) was positive for *M. ulcerans* DNA and the estimated bacterial load of this sample was low (10^2^–10^3^ organisms/gram).

Mapping of the samples collected in Point Lonsdale revealed that *M. ulcerans* DNA could be detected throughout Point Lonsdale and did not appear to be concentrated in one particular area or limited to one particular point source (Fig. 3). However, in Barwon Heads, positive faecal samples were only detected in the southern part of the town (data not shown). No seasonal trends were observed, with the number of positive samples, and the estimated bacterial loads of those samples, consistent between summer, autumn, winter and spring (data not shown).

All attempts at culturing *M. ulcerans* from possum faeces were unsuccessful. PCR-positive and PCR-negative possum faeces were inoculated into MGIT and onto Brown and Buckle and 7H10 slopes with antibiotics. The MGIT broths and Brown and Buckle slopes exhibited extensive fungal contamination after two weeks and were discarded. Despite the absence of fungal contamination on the 7H10 slopes, no growth of *M. ulcerans* was detected after 16 weeks incubation.

### Capture and examination of possums from Point Lonsdale

Over a 20-month period from February 2008 to November 2009, 42 ringtail possums and 21 brushtail possums were captured in Point Lonsdale and examined for BU disease. Among the ringtail possum cohort, 16 (38%) animals had laboratory-confirmed (PCR ± culture) *M. ulcerans* lesions and/or *M. ulcerans* PCR-positive faeces. Of the 11 animals with BU disease, nine had *M. ulcerans* PCR-positive faeces, one had *M. ulcerans* PCR-negative faeces and we were unsuccessful in collecting a faecal sample from the remaining animal (Table 3). Notably, five of the ringtail possums that did not have BU skin lesions had *M. ulcerans* PCR-positive faeces. Interestingly, as shown in Table 3, there was little difference in the median estimated bacterial loads of faeces from animals with BU skin lesions and animals without BU lesions. The incidence of *M. ulcerans* infection among the 21 brushtail possums was lower. One animal had a BU skin lesion and *M. ulcerans* PCR-positive faeces (estimated bacterial load, 10^3^–10^4^ organisms/gram) and four animals without BU lesions were found to be shedding low levels of *M. ulcerans* DNA in their faeces (10^2^ organisms/gram) (Table 3).

**Table 3:** *Mycobacterium ulcerans* status of ringtail and brushtail possums captured in Point Lonsdale, Victoria, and examined for BU lesions and the presence of *M. ulcerans* DNA in faeces.

*M. ulcerans* status of possums^a^	No. possums (median estimated bacterial load/gram faeces)		Total possums
	Ringtail	Brushtail	
BU lesions present; positive faeces	9 (10^5^–10^6^)	1 (10^4^–10^5^)	10
BU lesions present; negative faeces	1	0	1
BU lesions present; no faeces collected	1	0	1
BU lesions absent; positive faeces	5 (10^5^–10^6^)	4 (10^2^–10^3^)	9
BU lesions absent; negative faeces	26	16	42
Total	42	21	63

a *M. ulcerans* status refers to the presence or absence of external BU lesions (confirmed by PCR ± culture) and *M. ulcerans* DNA in faeces (detected by PCR).

The most common site for BU lesions was the tail (Fig. 2E). Amongst the 12 possums with BU disease, nine had lesions on the tail and four had lesions on the toe/foot (Table 4). Five of the ringtail possums had multiple lesions, with one animal having severe ulcerative and oedematous lesions on her nose (Fig. 2F), left upper lip, both fore paws, right hock, left hind leg and tail. Three of these animals were euthanased and full necropsies performed to determine the extent of the *M. ulcerans* infection. The results of these necropsies, along with the results of the other clinical samples taken from all 63 possums captured (including blood, buccal swabs and nasal swabs and urine), are described in a separate report (manuscript in preparation).

**Table 4:** Characteristics of possums with laboratory-confirmed BU lesions captured in Point Lonsdale, Victoria, 2008–09.

*ID*	*Species*	*Sex*	*Age*	*Site of BU lesion(s)* a
2	Ringtail possum	Female	Adult	Tail^b^ and toe^b^
9	Ringtail possum	Male	Adult	Tail
20	Ringtail possum	Male	Adult	Tail^b^
23	Ringtail possum	Male	Adult	Tail
30	Ringtail possum	Male	Juvenile	Hind foot
32	Ringtail possum	Female	Adult	Multiple ulcerative and oedematous lesions^b^^,^ c
46	Ringtail possum	Male	Adult	Tail
47	Ringtail possum	Male	Adult	Tail^b^
49	Brushtail possum	Female	Adult	Toe^b^
57	Ringtail possum	Female	Adult	Tail^b^ and ear
61	Ringtail possum	Male	Adult	Tail^b^, nose, arm and face/cheek
62	Ringtail possum	Female	Adult	Tail, nose and eye

a All lesions confirmed by PCR ± culture.

b Culture confirmed.

c Nose, tail, (R) hock, (L) hind leg, (L) front hand, (L) upper lip, (L) hind leg muscle.

### VNTR/MIRU typing of possum faecal samples demonstrates identity with human outbreak strain

**Figure 4:**
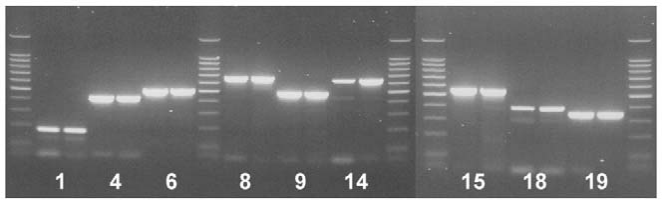
Variable number tandem repeat (VNTR) typing of *M. ulcerans* DNA in possum faeces demonstrates identity with human outbreak strain. Numbers represent VNTR loci [27]. At each locus: left PCR product, Victorian human patient isolate; right PCR product, DNA extracted from brushtail possum faeces collected in Point Lonsdale.

The two multiplex real-time PCR assays used in this study to detect *M. ulcerans* in environmental samples distinguish between *M. ulcerans* and other MPM that also harbour IS*2404* and IS*2606* [26]. However, we also sought to determine whether the DNA detected in environmental samples was from the same strain of *M. ulcerans* that causes disease in humans in Victoria. PCR reactions for 10 VNTR loci and three MIRU loci were performed on a subset of DNA extracts from possum faeces (estimated bacterial load 10^5^–10^6^ organisms/gram), aquatic plant biofilm (estimated bacterial load 10^3^–10^4^ organisms/gram) and water filters (estimated bacterial load 10^3^–10^4^ organisms/filter). The concentration of *M. ulcerans* DNA in the other sample types (for example, soil) has previously been shown to be insufficient for PCR amplification of these single copy loci [35]. DNA extracted from the possum faeces generated PCR amplicons of the same size (Fig. 4) and sequence as the Victorian human outbreak strain at all loci. As predicted by the lower concentration of *M. ulcerans* DNA in the samples, DNA extracts from the aquatic plant biofilm and water filter generated PCR amplicons at one locus only (VNTR locus 6 and 19, respectively). In each case the sequence was identical to the Victorian outbreak strain. These data provide evidence that the strain of *M. ulcerans* detected in these samples is the same as the strain which causes disease in humans in this region. The results also confirm that this method of analysis can only be applied successfully to samples (clinical or environmental) with an estimated *M. ulcerans* load of ≥10^5^ organisms/gram and should only be used as a confirmatory/epidemiological tool and not as the primary method by which all environmental samples are screened for the presence of *M. ulcerans* DNA [35].

### Whole genome sequencing of an *M. ulcerans* isolate from a ringtail possum

Illumina high-throughput short-read sequencing was used to compare the genome of an *M. ulcerans* isolate from a ringtail possum captured in Point Lonsdale (*M. ulcerans* JKD8170) and a human clinical isolate from Point Lonsdale (*M. ulcerans* JKD8049) obtained during the period of the *M. ulcerans* outbreak. This process generated 31,028,581 reads for JKD8170 and 10,921,914 reads for JKD8049. Bioinformatic analysis involved read mapping to the reference genome *M. ulcerans* Agy99 and reciprocal comparisons to consensus sequences derived from *de novo* sequence assemblies of each data set. These analyses revealed that both the possum and human isolates shared 5455 SNP differences compared to the reference genome (an African strain) but were differentiated from each other by only two SNPs (confirmed by PCR and Sanger DNA sequencing) across 5.6 Mb of chromosomal DNA sequence. These data confirm the extremely close genetic relationship between the human and possum isolates.

## Discussion

Elucidation of the mode of transmission and environmental reservoir(s) of *M. ulcerans* is essential for the development of strategies to control and prevent BU outbreaks. Early epidemiological studies from Uganda in the 1970s suggested that *M. ulcerans* may be associated with certain grasses growing at the edges of permanent swamps [36], [37], and that transmission to humans was via contact with this environmental source. However, attempts to culture *M. ulcerans* from a range of plants were unsuccessful [38]. The possible role of rodents in the ecology of *M. ulcerans* was also considered over 30 years ago [39], however the presence of the organism in the organs of 700 animals from a BU endemic area in Uganda could not be confirmed by culture. The development of IS*2404* PCR in the 1990s [16], [40] enabled researchers to detect the DNA of *M. ulcerans* and other MPM in a range of different samples, leading to a renewed search for the environmental reservoir(s). The PCR detection of *M. ulcerans* DNA in waterbugs from Benin and Ghana [18] and subsequent culture of *M. ulcerans* from a waterbug [14], focussed the search to aquatic habitats. Currently, the prevailing dogma is that the environmental reservoir of *M. ulcerans* is an abiotic or biotic component of aquatic, rather than terrestrial, ecosystems. Indeed, numerous epidemiological and environmental studies support this view [5], [11], [12], [14], [15], [17]–[21], [26], [41]–[43], including some of the data from our current study. We found that *M. ulcerans* could be detected in various aquatic samples including aquatic plants, biofilm and residue from filtered water (Table 1). The major strength of our study, however, was the use of a suite of real-time PCR assays targeting multiple regions in the *M. ulcerans* genome which, in addition to being highly sensitive, specific and less prone to contamination than conventional gel-based PCR [12], [13], enabled us to estimate the relative numbers of *M. ulcerans* in the various samples tested by determining the relative concentrations of *M. ulcerans* DNA among the different sample types.

By following this gradient of *M. ulcerans* DNA, we discovered that the faeces of two marsupial mammals (ringtail and brushtail possums), contained higher concentrations of *M. ulcerans* DNA than the other samples tested. The large-scale testing of possum faeces in BU high-, low- and non-endemic sites, and the subsequent capture and examination of possums in Point Lonsdale, generated a number of important findings. Firstly, we discovered that there is a high density of ringtail possums throughout Point Lonsdale that are excreting copious amounts of faeces, almost half of which are estimated to contain *M. ulcerans*, into the environment (Table 2, Fig. 3). Secondly, we observed a strong positive correlation between the BU endemicity of an area and the proportion and DNA concentration of *M. ulcerans*-positive possum faeces, with 41% of faecal samples collected in Point Lonsdale testing positive for *M. ulcerans* compared with less than 1% of faecal samples collected from non-endemic areas (p<0.0001). Similar results were obtained in Benin with a correlation between BU endemicity in patients and environmental results. Environmental studies detected variations in *M. ulcerans* DNA positivity rates of aquatic insects over time, and these changes were reflected in corresponding alterations of frequency of BU patients in the same foci [44]. Thirdly, 38% of captured ringtail possums and 24% of captured brushtail possums were found to have laboratory-confirmed *M. ulcerans* skin lesions, mostly on the tail or feet, and/or *M. ulcerans* PCR positive faeces (Table 3). One explanation for the observation that most lesions occurred on the extremities is that these sites have lower temperatures favouring the growth of *M. ulcerans*. Another possibility is that, because these sites have less fur, they are more susceptible to insect bites or skin trauma via contact with vegetation or fighting with other possums, which may lead to inoculation of *M. ulcerans*. Fourthly, we observed that five of the 14 ringtail possums, and four of the five brushtail possums, that were shedding *M. ulcerans* DNA in their faeces did not have BU skin lesions, indicating that the presence of *M. ulcerans* DNA in faeces is not limited to clinically diseased animals (Table 3). However, we noted that animals with multiple lesions tended to have higher estimated faecal loads of *M. ulcerans* than animals with single lesions (data not shown). Finally, whole genome sequencing confirmed the extremely close genetic relationship between the human and possum isolates.

Taken together, these findings suggest that possums may be an environmental reservoir for *M. ulcerans* in south-eastern Australia. If so, the biology of possums prompts a new interpretation/understanding of the life cycle of *M. ulcerans*. In particular, ringtail possums are exclusively arboreal, feeding on a variety of leaves of both native and introduced plants, as well as flowers and fruits [45], hence are unlikely to be exposed to *M. ulcerans* in soil or water. They are also caecotrophic. Caecotrophy is the ingestion of soft faeces of high nutritive value derived from caecal contents and is a critical factor in the ringtail possum's ability to utilise eucalypt foliage as a whole or major food source [46]. This behaviour may also favour gastrointestinal persistence of *M. ulcerans*. Brushtail possums are semi-arboreal, spending a considerable portion of their foraging time on the ground and, although mainly folivorous, have a more varied diet than ringtail possums [45]. The ecology of these species, which occur in strictly terrestrial habitats, contradicts the idea that the environmental host(s) of *M. ulcerans* are likely to reside primarily in aquatic environments, although the presence of *M. ulcerans* in aquatic habitats within the same location is also likely, based on data presented in this study. Thus, in light of our data, we suggest that reservoir species could include terrestrial mammals, and that the association of the disease with low-lying, wetter areas might be driven by the dependence of a vector species (such as mosquitoes [47]) on moist habitats.

**Figure 5:**
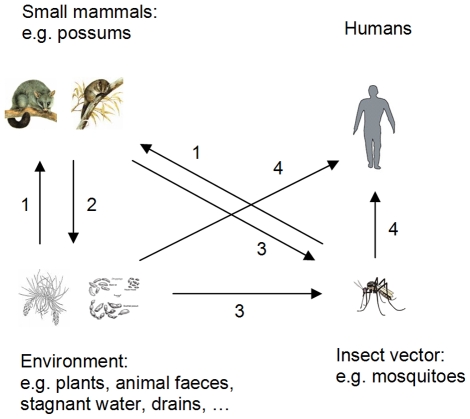
Proposed transmission pathways of *M. ulcerans* between the environment, mosquitoes, possums and humans. 1. Possums ingest *M. ulcerans* from the environment and/or infected by an insect vector. 2. Possums amplify and shed *M. ulcerans* into the environment. 3. Insect vectors become contaminated with *M. ulcerans* from the environment and/or from contact with infected possums. 4. *M. ulcerans* transmitted to humans via insect vector and/or direct contact with contaminated environment.

A disease reservoir may be defined as: “one or more epidemiologically connected populations or environments in which a pathogen can be permanently maintained and from which infection can be transmitted to the target population. Populations in a reservoir may be the same or a different species as the target and may include vector species” [48]. Our findings from Point Lonsdale suggest that at least one free-ranging mammal species (the ringtail possum), which can be very abundant in urban environments, forms part of a transmission cycle (Fig. 5) for *M. ulcerans* that could explain human outbreaks of BU in south-eastern Australia, although they may not necessarily be true maintenance hosts (that is, be able to maintain the organism in the absence of other environmental sources). However, bovine tuberculosis, caused by *Mycobacterium bovis*, and Johne's disease, caused by *Mycobacterium avium subsp. paratuberculosis*, are both maintained in wildlife reservoir species. In the United Kingdom, badgers (*Meles meles*) contribute to the spread of *M. bovis* between herds of cattle [49]. In New Zealand, where bovine tuberculosis is a major problem, the principle wildlife host for *M. bovis* is the common brushtail possum, which was originally imported from Australia and now occurs at such a high population density that it is a major agricultural and conservation pest [49].

The way in which *M. ulcerans* might be transmitted from an animal to humans is not clear. A similar epidemiology to leptospirosis, the most common zoonosis worldwide [50], in which rodents are reservoirs but the disease is acquired by contact with contaminated water, should be considered. We envisage that the transmission pathway for *M. ulcerans* may involve vegetation, vertebrate hosts and invertebrate vectors in both terrestrial and aquatic ecosystems (Fig. 5). Such a model represents a fundamental change to the existing views on the ecology of *M. ulcerans*, although the idea that *M. ulcerans* is not confined to low-lying swampy areas is not new [36]–[39], [51], [52]. While we lack important information about whether mosquitoes are productive or simply mechanical vectors, and have only limited information on the site of carriage/colonisation, either on or within mosquitoes, a number of lines of evidence implicate mosquitoes as vectors of *M. ulcerans* in Victoria [6], [53]–[55]. Given that we found active *M. ulcerans* lesions in 26% of captured ringtail possums, transmission to humans might occur when an adult mosquito that has fed on a diseased possum, or rested on vegetation contaminated by a possum lesion, subsequently bites a human. Another possibility is that heavy environmental contamination with possum faeces containing *M. ulcerans* would enable mosquitoes (either as larvae or adults) to come into contact with *M. ulcerans*, in contaminated soil/water in roof gutters or drains (Fig. 5). This is supported by a study by Tobias *et al.* which showed that, in a feeding experiment where mosquito larvae were fed possum faecal material spiked with *M. ulcerans* or *M. marinum*, *M. ulcerans* accumulated within the mouth and midgut whereas *M. marinum* did not [55].

Key to determining which of these potential routes of transmission is most likely (or possible) is the demonstration of viable *M. ulcerans* organisms in possum faeces. We acknowledge that the detection of *M. ulcerans* DNA in possum faeces does not necessarily indicate the presence of viable organisms. However we, like many others who have attempted to culture *M. ulcerans* from environmental samples [14], have currently been unable to culture *M. ulcerans* from possum faeces. This was despite the fact that some of the samples had real-time PCR signals equivalent to those obtained for the lesion swabs from which culture of *M. ulcerans* was successful (data not shown). We believe that this has been largely due to the presence of fungi or fungal spores in the faecal samples which, despite decontamination methods, rapidly grew in broth cultures and on Brown and Buckle slopes and inhibited the growth of slower growing organisms such as *M. ulcerans*. Furthermore, on the basis of subsequent real-time PCR studies, it has become evident that the organisms are tightly associated with the particulate matter and that homogenising faeces in bead bottles results in very few bacteria in the suspension that would normally be used to inoculate the culture media (C. O'Brien, unpublished). We have also found that intact DNA can be recovered from possum faeces many months after sampling and that DNase treatment of the faecal homogenate does not lead to a reduction in the PCR signal (data not shown). This suggests that intact *M. ulcerans* organisms are present (though not necessarily viable), rather than just free *M. ulcerans* DNA.

There is also the question of whether mammals could act as reservoirs in sub-Saharan Africa, where the majority of BU cases occur. Recent studies in Ghana failed to detect *M. ulcerans* in the organs or faeces of rodents and shrews [17], [56]. However these authors did not reject the hypothesis that these, or other species of small terrestrial mammals, may be part of the reservoir of *M. ulcerans* in this setting. Recent work conducted by our group, including the post-mortem examination of ringtail possums and rats (*Rattus rattus*) with and without clinical BU disease, has shown that *M. ulcerans* can be present in the gastrointestinal tracts of animals but not in the organs of the same individual (manuscript in preparation). We are currently investigating the potential role of other mammal species as hosts for *M. ulcerans* in the Australian setting.

This study has led to a major a shift in our understanding of the environmental distribution of *M. ulcerans* in south-eastern Australia. It is hoped that the results presented here, along with our continuing laboratory and field research, will take us closer to elucidating the mode of transmission and environmental reservoir(s) of *M. ulcerans* and in turn the development of strategies to control and prevent this important yet often neglected human disease.

## References

[1] George K, Pascopella L, Welty D, Small P (2000). A *Mycobacterium ulcerans* toxin, mycolactone, causes apoptosis in guinea pig ulcers and tissue culture cells.. Infect Immun.

[2] World Health Organization (2008). Buruli ulcer: progress report, 2004–2008.. Wkly Epidemiol Rec.

[3] MacCallum P, Tolhurst J, Buckle G, Sissons H (1948). A new mycobacterial infection in man.. J Path Bacteriol.

[4] Francis G, Whitby M, Woods M (2006). *Mycobacterium ulcerans infection*: a rediscovered focus in the Capricorn Coast region of central Queensland.. Med J Aust.

[5] Johnson PD, Veitch MG, Leslie DE, Flood PE, Hayman JA (1996). The emergence of *Mycobacterium ulcerans* infection near Melbourne.. Med J Aust.

[6] Johnson PDR, Azuolas J, Lavender CJ, Wishart E, Stinear TP (2007). *Mycobacterium ulcerans* in mosquitoes captured during an outbreak of Buruli ulcer, southeastern Australia.. Emerg Infect Dis.

[7] Mitchell PJ, Jerrett IV, Slee KJ (1984). Skin ulcers caused by *Mycobacterium ulcerans* in koalas near Bairnsdale, Australia.. Pathology.

[8] Portaels F, Chemlal K, Elsen P, Johnson PD, Hayman JA (2001). *Mycobacterium ulcerans* in wild animals.. Rev Sci Tech.

[9] van Zyl A, Daniel J, Wayne J, McCowan C, Malik R (2010). *Mycobacterium ulcerans* infections in two horses in south-eastern Australia.. Aust Vet J.

[10] Elsner L, Wayne J, O'Brien CR, McCowan C, Malik R (2008). Localised *Mycobacterium ulcerans* infection in a cat in Australia.. J Feline Med Surg.

[11] Marsollier L, Robert R, Aubry J, Saint Andre J, Kouakou H (2002). Aquatic insects as a vector for *Mycobacterium ulcerans*.. Appl Environ Microbiol.

[12] Williamson HR, Benbow ME, Nguyen KD, Beachboard DC, Kimbirauskas RK (2008). Distribution of *Mycobacterium ulcerans* in Buruli Ulcer Endemic and Non-Endemic Aquatic Sites in Ghana.. PLoS Negl Trop Dis.

[13] Benbow ME, Williamson H, Kimbirauskas R, McIntosh MD, Kolar R (2008). Aquatic invertebrates as unlikely vectors of Buruli ulcer disease.. Emerg Infect Dis.

[14] Portaels F, Meyers WM, Ablordey A, Castro AG, Chemlal K (2008). First Cultivation and Characterization of *Mycobacterium ulcerans* from the Environment.. PLoS Negl Trop Dis.

[15] Ross B, Johnson P, Oppedisano F, Marino L, Sievers A (1997). Detection of *Mycobacterium ulcerans* in environmental samples during an outbreak of ulcerative disease.. Appl Environ Microbiol.

[16] Stinear T, Ross B, Davies J, Marino L, Robins-Browne R (1999). Identification and characterization of IS*2404* and IS*2606*: two distinct repeated sequences for detection of *Mycobacterium ulcerans* by PCR.. J Clin Microbiol.

[17] Vandelannoote K, Durnez L, Amissah D, Gryseels S, Dodoo A (2010). Application of real-time PCR in Ghana, a Buruli ulcer-endemic country, confirms the presence of *Mycobacterium ulcerans* in the environment.. FEMS Microbiol Lett.

[18] Portaels F, Elsen P, Guimaraes-Peres A, Fonteyne P, Meyers W (1999). Insects in the transmission of *Mycobacterium ulcerans* infection.. Lancet.

[19] Marsollier L, Stinear T, Aubry J, Saint Andre J, Robert R (2004). Aquatic plants stimulate the growth of and biofilm formation by *Mycobacterium ulcerans* in axenic culture and harbor these bacteria in the environment.. Appl Environ Microbiol.

[20] Marsollier L, Severin T, Aubry J, Merritt R, Saint Andre J (2004). Aquatic snails, passive hosts of *Mycobacterium ulcerans*.. Appl Environ Microbiol.

[21] Eddyani M, Ofori-Adjei D, Teugels G, De Weirdt D, Boakye D (2004). Potential role for fish in transmission of *Mycobacterium ulcerans* disease (Buruli ulcer): an environmental study.. Appl Environ Microbiol.

[22] Stinear T, Seemann T, Pidot S, Frigui W, Reysset G (2007). Reductive evolution and niche-adaptation inferred from the genome of *Mycobacterium ulcerans*, the causative agent of Buruli ulcer.. Genome Res.

[23] Stinear T, Johnson PDR (2007). From Marinum to Ulcerans: a Mycobacterial Human Pathogen Emerges.. Microbe.

[24] Stinear T, Mve-Obiang A, Small P, Frigui W, Pryor M (2004). Giant plasmid-encoded polyketide synthases produce the macrolide toxin of *Mycobacterium ulcerans*.. PNAS.

[25] Triggs B (2004). Tracks, scats and other traces: a field guide to Australian mammals.

[26] Fyfe JA, Lavender CJ, Johnson PD, Globan M, Sievers A (2007). Development and application of two multiplex real-time PCR assays for the detection of *Mycobacterium ulcerans* in clinical and environmental samples.. Appl Environ Microbiol.

[27] Ablordey A, Swings J, Hubans C, Chemlal K, Locht C (2005). Multilocus Variable-Number Tandem Repeat Typing of *Mycobacterium ulcerans*.. J Clin Microbiol.

[28] Hilty M, Yeboah-Manu D, Boakye D, Mensah-Quainoo E, Rondini S (2006). Genetic diversity in *Mycobacterium ulcerans* isolates from Ghana revealed by a newly identified locus containing a variable number of tandem repeats.. J Bacteriol.

[29] Stragier P, Ablordey A, Meyers W, Portaels F (2005). Genotyping *Mycobacterium ulcerans* and *Mycobacterium marinum* by using mycobacterial interspersed repetitive units.. J Bacteriol.

[30] Rumble SM, Lacroute P, Dalca AV, Fiume M, Sidow A (2009). SHRiMP: accurate mapping of short color-space reads.. PLoS Comput Biol.

[31] Steen JA, Harrison P, Seemann T, Wilkie I, Harper M (2010). *Fis* is essential for capsule production in *Pasteurella multocida* and regulates expression of other important virulence factors.. PLoS Pathog.

[32] Zerbino DR, Birney E (2008). Velvet: algorithms for de novo short read assembly using de Bruijn graphs.. Genome Res.

[33] Australian Bureau of Statistics (2007). 2006 Census of Population and Housing.. http://www.abs.gov.au.

[34] Meyers WM, Shelly WM, Connor DH, Meyers EK (1974). Human *Mycobacterium ulcerans* infections developing at sites of trauma to skin.. Am J Trop Med Hyg.

[35] Lavender CJ, Stinear TP, Johnson PD, Azuolas J, Benbow ME (2008). Evaluation of VNTR typing for the identification of *Mycobacterium ulcerans* in environmental samples from Victoria, Australia.. FEMS Microbiol Lett.

[36] Barker DJ (1971). Buruli disease in a district of Uganda.. J Trop Med Hyg.

[37] Barker DJ, Clancey JK, Morrow RH, Rao S (1970). Transmission of Buruli disease.. Br Med J.

[38] Stanford J, Paul R (1973). A preliminary report on some studies of environmental mycobacteria.. Ann Soc Belg Med Trop.

[39] Revill WDL, Morrow RHJ, Parson W, Kiryabwire JWM, Shaper AG, Kibukamusoke JW, Hutt MSR (1972). *Mycobacterium ulcerans* infection (Buruli ulcer).. Medicine in a tropical environment.

[40] Ross B, Marino L, Oppedisano F, Edwards R, Robins-Browne R (1997). Development of a PCR assay for rapid diagnosis of *Mycobacterium ulcerans* infection.. J Clin Microbiol.

[41] Portaels F (1995). Epidemiology of mycobacterial diseases.. Clin Derm.

[42] Stinear T, Davies J, Jenkin G, Hayman J, Oppedisano F (2000). Identification of *Mycobacterium ulcerans* in the environment from regions in Southeast Australia in which it is endemic with sequence capture-PCR.. Appl Environ Microbiol.

[43] Uganda Buruli Group (1971). Epidemiology of *Mycobacterium ulcerans* infection at Kinyara, Uganda.. Trans R Soc Trop Med Hyg.

[44] Portaels F, Silva MT, Meyers WM (2009). Buruli ulcer.. Clin Dermatol.

[45] Kerle JA (2001). Possums : the brushtails, ringtails and greater glider.

[46] Hume ID (1989). Nutrition of marsupial herbivores.. Proc Nutr Soc.

[47] Clements AN (1992). The biology of mosquitoes.

[48] Haydon DT, Cleaveland S, Taylor LH, Laurenson MK (2002). Identifying reservoirs of infection: a conceptual and practical challenge.. Emerg Infect Dis.

[49] Biet F, Boschiroli ML, Thorel MF, Guilloteau LA (2005). Zoonotic aspects of *Mycobacterium bovis* and *Mycobacterium avium-intracellulare* complex (MAC).. Vet Res.

[50] Adler B, de la Pena Moctezuma A (2009). Leptospira and leptospirosis.. Vet Microbiol.

[51] Barker DJ (1973). Epidemiology of *Mycobacterium ulcerans* infection.. Trans R Soc Trop Med Hyg.

[52] Barker DJ, Ninkibigaya V (1972). Buruli disease and patients' activities.. East Afr Med J.

[53] Johnson P, Lavender C (2009). Correlation between Buruli ulcer and vector-borne notifiable diseases, Victoria, Australia.. Emerg Infect Dis.

[54] Quek TY, Athan E, Henry MJ, Pasco JA, Redden-Hoare J (2007). Risk factors for *Mycobacterium ulcerans* infection, southeastern Australia.. Emerg Infect Dis.

[55] Tobias NJ, Seemann T, Pidot SJ, Porter JL, Marsollier L (2009). Mycolactone gene expression is controlled by strong SigA-like promoters with utility in studies of *Mycobacterium ulcerans* and Buruli ulcer.. PLoS Negl Trop Dis.

[56] Durnez L, Suykerbuyk P, Nicolas V, Barriere P, Verheyen E (30 April 2010). The role of terrestrial small mammals as reservoir of *Mycobacterium ulcerans* in Benin.. Appl Environ Microbiol.

